# Digital health promotion: promise and peril

**DOI:** 10.1093/heapro/daab134

**Published:** 2021-12-12

**Authors:** Amanda Koh, De Wet Swanepoel, Annie Ling, Beverly Lorraine Ho, Si Ying Tan, Jeremy Lim

**Affiliations:** 1 Department of Economics, National University of Singapore, 21 Lower Kent Ridge Road, Singapore 119077, Singapore; 2 Department of Speech Pathology and Audiology, University of Pretoria, Lynnwood Rd & Roper Street, Pretoria, 0001, South Africa; 3 Ear Sciences Centre, School of Surgery, The University of Western Australia, 35 Stirling Hwy, Crawley WA 6009, Perth, Australia; 4 Ear Science Institute Australia, 2/1 Salvado Rd, Subiaco WA 6008, Australia; 5 Saw Swee Hock School of Public Health, National University of Singapore, 12 Science Drive 2, #10-01, 117549, Singapore; 6 Department of Health, Health Promotion Bureau, San Lazaro Compound, Tayuman, Sta. Cruz, Manila 1003, Philippines; 7 Saw Swee Hock School of Public Health, National University of Singapore, 21 Lower Kent Ridge Road, Singapore 119077, Singapore

**Keywords:** digital health, digitalization, personalized health, health behaviors, community health promotion

## Abstract

The World Health Organization defines health promotion as process of enabling people to increase control over their health and its determinants, and thereby improve their health. As the world transitions into the information age, incorporating digital technologies into health promotion is becoming commonplace. This article discusses current applications of digital health promotion (DHP) and addresses its potential benefits, challenges, as well as how differences in cultures, governance models and digital readiness across the globe will shape the implementation of DHP differently in each society. The benefits include expanding access to health information and health promoting services, lowering scaling up costs, personalizing health advice and real-time ‘nudging’ toward healthier options. Key challenges would involve privacy control, appropriate use of data including secondary usage beyond the original intention, defining the limits of ‘nudging’ and the right of free choice, and ensuring widespread accessibility and affordability to minimize the exacerbation of social inequities. Finally, we discuss the enabling factors for successful DHP implementation, suggesting measures that should be taken at both individual and system levels. At the individual level, we explore the factors necessary to access and benefit from DHP meaningfully; at the system level, we examine the infrastructure required to provide wide access, establish trust among users and enable sustainability of behavioral changes.

## Introduction

From smartphone applications that provide personalized health programs to activity trackers that monitor heart rates, technology is shaping nearly every aspect of our lives. As the world transitions from the industrial to information age, health promotion too, is progressing into a new technological era of digital health promotion (DHP). This article examines current concepts and applications of DHP; considers the potential to greatly improve outreach, enhance impact and reduce costs; and explores the potential challenges policy makers should consider. 

The use of digital media and digital products for health promotion has grown exponentially in the past two decades. This pace of growth has far outstripped the accumulation of evidence. For example, in a review of mobile app-based health promotion programs by Lee *et al.* (Lee *et al.*, 2018), only 12 randomized controlled studies were identified. In contrast, the consultancy IQVIA estimates that more than 200 health apps are being added daily, with over 318 000 health apps available in 2017 alone (Aitken *et al.*, 2017).

The World Health Organization defines health promotion as the ‘process of enabling people to increase control over their health and its determinants, and thereby improve their health’ (The Bangkok Charter for Health Promotion in a Globalized World, 2005). Health promotion not only involves personal agency through increasing knowledge of and adopting a healthy lifestyle but also includes an ecological overarching frame which focuses on providing social and other support, enabling access and increasing engagement.

As the term suggests, DHP can be seen as a branch extension of health promotion. It is a term used to encompass the applications of digital technologies to health promotion. With ever-improving technologies, penetration of connectivity and wider adoption of devices including smartphones and wearables, there is great potential for DHP. Global Systems for Mobile Communications Association (GSMA), the industry body for mobile operators, estimated that in July 2020, there were 8.8 billion mobile connections and 5.1 billion unique mobile device subscribers with year-on-year growth of 6.2% and 3.72%, respectively (GSMA, 2020). These devices represent a formidable resource for effective, personalized and accessible health communication that can be quickly scaled.

Digital technology has brought new tools in advancing population health, addressing a wide range of access and cost challenges. Not only do these technologies enable health promoters to reach hard-to-access populations more easily and affordably, often in the home or community setting rather than formal healthcare facilities, it also allows for targeting of different forms of health communication to specific sub-populations, and even personalization to individuals.

We discuss in the following sections individual and population health DHP considerations, enabling factors for successful DHP adoption and address its potential benefits, challenges, as well as how differences in cultures, governance models and digital readiness across the globe will shape the implementation of DHP in each society.

## Digital health promotion—individual and population health considerations

Digitalization permits data about an individual to be captured from multiple sources including wearable sensors and the environment around the individual. Combined with other data on disease states, personality profile, social preferences and health goals, this information is then synthesized, analyzed typically using sophisticated machine learning algorithms and converted into insights as to what nudges or interventions would be most appropriate for a given individual at a specific place and time. These data are important for providers to design DHP interventions and to make decisions on resource allocations.

Besides providing for inexpensive means to access individuals and influence behavioral change, digitization allows for aggregation of individual experiences at the population level, allowing policy planners granular visibility of potential health promotion measures and their impact at a population level. These population level findings can be used to improve existing measures, identify unreached groups and plan adjacent services such as facilities for in-person interventions.

## Enabling digital health promotion

**Fig. 1:**
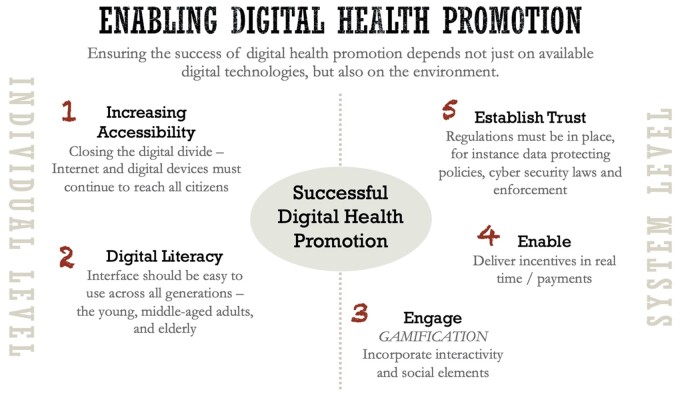
Ecological system view of enabling digital health promotion.

Ensuring the success of DHP depends not just on available digital technologies, but also on the socio-economic, political and governance environments. Figure 1 summarizes our view of the five key aspects for successful DHP, which are divided into individual (increasing accessibility, ensuring digital literacy), and systems level (engaging, enabling, and establishing trust).

At the individual level, governments need to look at increasing accessibility and affordability. DHP providers should ensure the digital interface is simple and straightforward so as to appeal to a wider target audience, including those who may not be digitally literate initially. Minimizing the current digital divide is key to ensuring a successful DHP at population levels. With the decreasing cost of digital technologies (Effy *et al.*, 2018), aiming to reach all citizens digitally for health promotion even for lower- to middle-income countries will be possible. Even historically marginalized groups, like persons with disabilities in low- and middle-income countries, have high rates of mobile phone ownership and perceive the devices as enabling access to services (GSMA, 2019a,b). However, the gender gap merits specific attention. Women in lower- to middle-income countries are 10% less likely than men (197 million women) to own a mobile and 23% less likely to access mobile services. Advocates have a responsibility in enabling the upstream determinants so that all individuals and communities can meaningfully benefit from DHP.

At the systems level, we have to go beyond access to devices and connectivity. DHP leaders should focus also on engaging, enabling and crucially, establishing trust between users and organizations, particularly regarding the use of personal data.

Engaging users can come in the form of incorporating interactivity and social elements through gamification. For instance, a recent systematic review on exergaming involving 1520 elderly persons found improvements in balance and mobility (Pacheco, 2020) and the interactivity was deemed an important element in achieving these results.

Next, offering and delivering incentives in real time is an example of enabling users to pursue and maintain good health. A good example would be Singapore’s H365 application, having successfully incorporated a multi-tiered, sure-win reward system that encouraged individuals to kickstart and sustain behavior change. Other examples include Vitality (AIA Singapore, 2020) created by South Africa’s Discovery (Shapshak, 2020), and used around the world by various insurers.

Lastly, establishing trust involves directing users to reliable and comprehensible health information found on digital platforms, as well as implementing data protection policies to establish accountability and ensure data privacy, especially since DHP will increasingly deal with sensitive personal health data.

## Potential benefits of digital health promotion

### Expanding access and targeting at-risk populations

With online platforms such as YouTube and Facebook as well as their Chinese counterparts becoming ubiquitous, DHP by tapping on the same platforms will also be able to significantly expand its access and reach. In a recent study published in MIS Quarterly, researchers have found that YouTube alone contains more than 20 000 healthcare-related videos (Stein, 2020). These are produced by individual users, as well as established organizations such as the Mayo Clinic, the American Diabetes Association and the American Nutrition Association (Medical Xpress, 2020).

With one in three adults living with at least one chronic disease (Hajat and Stein, 2018), technologies that enable massive scale will be vital in ameliorating the shortage of health professionals. Even in rural areas, 4G connectivity is often available (one out of three of India’s 4G subscribers live in villages—Aneja, 2018), and loading relevant public health content on these consumer technology platforms can allow reach where conventional health promotion has previously found it difficult to penetrate due to distance, language and cultural barriers.

Besides its potential to access remote or hard-to-reach communities, DHP also permits targeting of vulnerable individuals who may have difficulty seeking medical help due to stigma or out-of-pocket expenses. The use of online platforms can offer a discrete and anonymous method for seeking support, providing those who tend to shy away from conventional therapy or counseling additional privacy. Amid the COVID-19 pandemic and resultant lockdowns in various parts of the world, the rise of mental health issues has led many to online platforms to seek support. In India, online mental wellness applications such as YourDOST, Wysa and Mfine have seen a 30% increase in the number of online appointments and call-ins made (Bansal, 2020), with COVID-19-induced anxiety major contributors to this. This has also been the experience in other highly stigmatized diseases such as HIV/AIDS, where patients have preferred to seek social support from various online platforms (Reeves, 2001).

Furthermore, digitalization increasingly enables health promotion to leverage the power of social media—a platform that offers direct access to existing online social networks. With social media, communities that are often harder to reach through traditional health promotion such as those of low socio-economic status, young people and ethnic minorities, can now be targeted (Moorhead *et al.*, 2013). For instance, Facebook—a social media platform that adults, especially middle-aged ones frequent—has invested resources in suicide prevention programs (Davis, 2019). Through the use of AI, Facebook identifies posts that express suicidal thoughts (Muriello *et al.*, 2018). Once identified, the system directs these posts to trained members of the Community Operations team who determine whether the posters are at-risk individuals, thereby providing them with resources and relevant support options. A parallel program is offered by Facebook-owned Instagram.

### Lowered upscaling costs

The incremental cost of scaling in digital health interventions is minimal in terms of technology, but the potential impact is tremendous. Once a software is designed and the underlying technology platform sufficient for scale, it can be used repeatedly to reach millions of targeted individuals. In essence, the bulk of the cost is upfront in the creation of the technology, with minimal upscaling costs. Health promotion agencies today can and should leverage on existing high-reach consumer technology platforms such as Facebook, WeChat and so on to reach large populations with less effort and expense compared to recreating distribution and outreach channels.

Aside from lowering upscaling costs, effective DHP can also reduce pressures on the healthcare system. A recent study by IQVIA has estimated that the adoption of DHP applications in just five patient populations alone would save the United States healthcare system US$7 billion per year (Aitken *et al.*, 2017), amounting to about 1.4% of total costs. These cost-savings are estimated based on five high priority Digital Health app categories which included (i) Diabetes Prevention, (ii) Diabetes, (iii) Asthma, (iv) Pulmonary Rehabilitation (PR) and (v) Cardiac Rehabilitation. For instance, for Diabetes Prevention, a long-term decrease in diabetes incidence led to significant cost-savings with reduced physician visits and medical prescriptions. PR also saw cost-savings given the lower cost of app-based PR as compared to face-to-face PR.

In addition, DHP is not only able to target millions without requiring additional spending on manpower in the process, but costs involved in software development are also much less than traditional therapeutics. For instance, many digital therapeutics companies such as Second Nature required less than $5 million (Beckwith, 2018) to develop their technologies. The first ever digital behavioral change program to be recognized by the United Kingdom’s National Health Service, Second Nature targets weight-loss and the cultivation of healthy habits such as more regular exercise, better nutrition etc (Second Nature, 2020). Incorporating a technology package to its program, Second Nature uses smart scales and a steps tracker to measure progress, maintain motivation, and record success.

However, it should be acknowledged that system-level costs such as start-up and investment costs related to the technical operations of DHP, especially in low- and middle-income countries, could be significant. Hence, the upscaling of DHP interventions in these countries might be more challenging than high-income countries.

### Personalized public health

In health promotion, one size does *not* fit all. With ongoing data capture and further learning, personalization of health promotion interventions can be more effective and efficient, establishing a virtuous cycle. Additionally, personalization can also reduce confusion and anxiety for individuals over receiving conflicting recommendations (Esserman *et al.*, 2017). A possible roadmap for this might be moving away from national health promotion plans to smaller more targeted interventions focused on specific communities both geographic and virtually connected and finally ever-more customization toward the individual.

In recent years, more studies are emerging that support the benefits of personalization. In one study on dietary intake and weight, researchers showed that self-monitoring diets including mobile app-based food logging, and provision of tailored feedback was effective in inducing positive behavior change. Over 6 months, participants reduced their intake of energy-dense and nutrient-poor foods, facilitating weight loss and reducing future chronic disease risk (Kerr *et al.*, 2016). Besides, digitally delivered coaching has been shown also to improve outcomes in cardiovascular disease. Data from the HATICE study demonstrated that people age 65 years or above at increased risk of cardiovascular disease were provided a digitally delivered coach-supported self-management program had small but sustained improvements in risk factors over an 18-month period compared to controls (Richard *et al.*, 2019).

Personalization is applicable to health screening too. In the WISDOM study, women with different genetic risk profiles were enrolled into different screening regimes (Esserman *et al.*, 2017). Today, it is typical that virtually everyone is deemed to have an average risk and hence undergo health checks routinely. Or at most individuals would be classified into low-, average- and high-risk categories with three screening pathways. In another example, The Lancet Commission on Dementia recently found hearing loss in mid-life to be the most important modifiable risk factor for dementia and recommend promotion of hearing aids as preventative strategy. DHP can target at-risk individuals with targeted messaging and resources. In the future, better understanding of risk factors including at the molecular level can enable health systems to offer more personalized screening and better use of health resources (Livingston *et al.*, 2020).

Even though health and fitness mobile applications are proliferating, there is a need to be more prudent in evaluating their efficacies or effectiveness before deployment. Whilst it is heartening developers are paying attention to health, the challenge lies in the fact that many of these applications are not evidence-based and are of poor information quality (Grady *et al.*, 2018). Applications are selected based on perceived relevance, brand recognition and user ratings, rather than evidence of effectiveness. Given the large number of applications, it is a major challenge to assess them rigorously and hence, many applications are released to the public without being effectively trialled and tested (Cook *et al.*, 2016). To help guide the public, both the UK National Health Service and US National Health Institute have funded online libraries of evidence-based and publicly endorsed health apps (One You, 2020). However, these cover over a fraction of the available apps.

### Increasing receptivity: engaging and motivating individuals through real-time nudging toward healthier options as well as incorporating social elements

Beyond greater access, reduced costs and deeper personalization, DHP also has the potential to increase the level of engagement and receptivity of its target audience through ‘nudging’. Nudging as defined by Thaler and Sunstein in their popular 2008 book ‘*Nudge—Improving Decisions about Health, Wealth and Happiness*’ as ‘any aspect of the choice architecture that alters people’s behavior in a predictable way without forbidding any options or significantly changing their economic incentives’ (Thaler and Sunstein, 2008). Thaler and Sunstein provided numerous examples of population level nudges such as placing healthier foods at eye level or sending reminders to citizens that they were in the very small minority that had not paid their taxes. These were effective in and of themselves, but augmented by technology, can be much more personalized and powerful or as Yeung terms ‘hypernudges’ (Yeung, 2017).

Many of us carry our smartphones with us everywhere we go and the embedded sensors enable a smartphone to create a profile of its user, foresee his or her needs, then make recommendations via push notifications accordingly (MIT Technology Review, 2020). These technologies and applications can be purposed for DHP, using physiological data captured by wearables complemented by geo-spatial data to provide real-time suggestions for users, thereby enhancing engagement and inexpensively motivating individuals to pursue good health.

Humans are social creatures, hard-wired for interaction and engaging with one another in day-to-day activities (Castiello *et al.*, 2010). Interdependent beings, individuals are highly receptive to social relationships. As such, DHP has the potential to appeal to many through incorporating social elements into its hardware. Not only will this increase the level of engagement and hence retention rates but also generate social support by providing encouragement for one another. As we are wired to connect with others especially with those in similar circumstances, the creation of online social networks allows patients to share crowd-sourced knowledge, enabling them to leverage on and learn from the personal experiences of many others across the world.

### Aggregated data enabling predictive epidemiology

At the system level, one benefit of digitalizing health promotion is the resultant ability to aggregate individual data points and build predictive models that can galvanize population and individual actions. From farm vehicle crashes (Ranapurwala *et al.*, 2019) to COVID-19 public health measures (Flaxman *et al.*, 2020; Hsiang *et al.*, 2020), predictive epidemiologic models have helped policy makers and individuals to sense make complex multi-factorial issues and distil into discrete options. The United Kingdom’s backing down from a ‘herd immunity’ strategy after publication of the Imperial College Covid Response Team’s epidemiological models is a well-known case in point (Raphael, 2020).

## Challenges and peril

Despite the excitement around the benefits that DHP can bring, it remains a double-edged sword. Done well, it can provide improvements in the area of health promotion as illustrated by the examples provided above. Yet, it creates new challenges that public health agencies and governments will need to address.

### Privacy and secondary use conundrums

With more and more data, privacy issues especially with secondary data use can be problematic. For example, in 1997, blood samples collected for Australia’s national newborn screening (NBS) program that were stored in cards were used by the Western Australian police for the investigation of a case of suspected incest without the consent of children or the birth mothers (Bowman and Studdert, 2011).

Although the offender was eventually convicted, the hospital determined there was a substantial risk of losing the public’s trust in its screening program and parents consequently deciding not to have their children screened. Eventually, the hospital decided to erase decades’ worth of blood samples and samples are now kept for only 2 years after birth.

The highly sensitive nature of medical data and its potential for secondary use (and misuse) raise many concerns regarding data protection, especially for populations without specific privacy protections in place. To date, the governance of health data appears patchy, with only about half of WHO countries having data protection laws that govern personal health data (Vayena *et al.*, 2018). However, detailed guidance does exist with the OECD releasing in 2019 a health data governance framework encouraging the ‘availability and use of personal health data to serve health-related public interest’ whilst ‘promoting the protection of privacy, personal health data and data security’.

### Algorithmic bias

The algorithms which power many digital interventions are known to harbor bias and these may exclude specific populations or communities or inappropriately single them out. In 2019, American researchers found that a commercial algorithm assigned sicker black patients the same level of risk as white patients because the algorithm used health costs as a proxy for health needs. This proxy is grossly inappropriate of course given widely known disadvantages black populations face in accessing and affording healthcare in the USA but financial data is easier to obtain and use in training sets for machine learning. The researchers concluded that the algorithm wrongly assigned more than half the number of black patients requiring additional care (Obermeyer *et al.*, 2019). Algorithmic biases resulted from inherent data issue or machine learning picking up peculiar patterns that are not generalizable have been documented in cardiovascular clinical trials that favored men’s participations over women’s and research studies that neglected specific populace in the society (Luminary Labs, 2021). These scenarios imply that DHP interventions should always bear in mind the potential pitfalls of the deep learning predictions and account for possible negligence of certain demographics such as ethnicity, gender, age group and disease types in the promotion, assignment and prescription of interventions.

### Cybersecurity risks

Cybersecurity risks are not unique to healthcare but the healthcare sector is a prime target for criminals given the value of health data (Trend Micro, 2017). For instance, whilst Facebook’s suicide prevention program may have been useful in identifying and intervening automatically, it also necessarily identifies at-risk individuals whose personal details may be unveiled in the event of a cybersecurity incident. In September 2018, a large-scale data breach was exactly what happened, with the personal profiles of more than 50 million users compromised (Isaac and Frenkel, 2018). Although Facebook had not disclosed whether these compromised users who were in its suicide prevention algorithm, concerns were raised regarding Facebook’s use of user-data without consent, leaving privacy experts questioning whether Facebook can be considered a trustworthy platform especially in the handling of sensitive health data. While Facebook creates and stores new health information that deals with mental health issues in an attempt to prevent suicides, it must be taken into account that Facebook and other social media platforms in general do not need to uphold the same privacy standards (Goggin, 2019) as healthcare providers. Present health data protection laws such as the Health Insurance Portability and Accountability Act (HIPAA) in the USA do not extend to Facebook or its suicide prevention program and due consideration should be for all organizations that deal with personal health information to be regulated by equivalent standards whether or not they are healthcare organizations. Similar risk could also occur in DHP interventions that contain massive health data, personal data and financial data in different mobile apps and health information systems that represent either a niche population or general population, predisposing them to data breaches, cyberattacks and ransomware attacks.

### Misinformation and malicious falsehoods

Going digital enables responsible public health agencies to reach millions around the world quickly and inexpensively. The World Health Organization has 33.1 million followers on Facebook as of July 2020, whilst the Bill and Melinda Gates Foundation attracts 1.5 million followers. However, the same ease of reach applies also to misinformation and it is unsurprising that WHO Director General Tedros Adhanom Ghebreyesus declared in February 2020 that ‘We’re not just fighting an epidemic; we’re fighting an infodemic’ (Zarocostas, 2020).

Public health information can often be complex and messaging nuanced, as professionals are loath to over-simplify. But this can confuse the public or even worse, be wrongly perceived as part of a cover-up. The dengue vaccine disaster in the Philippines is a sobering illustration of how quickly confidence can be eroded. Between 2015 and 2018, the number of Filipino respondents ‘strongly agreeing’ vaccines are important dropped from 93% to 32%. The numbers strongly agreeing vaccines are safe fell from 82% to 21% and confidence in the effectiveness of vaccines plummeted from 82% to only 22% (Larson *et al.*, 2019). Health Under-Secretary Eric Domingo attributed the decline in public confidence at least partly to the proliferation of misinformation online, saying ‘Some of our countrymen have access to social media. When they see false information, this instils fears, and adds up to our problems in combating low vaccine coverage’ (Bernardo, 2019).

One year later, the Philippines was wrecked by a measles outbreak. The dengue vaccine missteps had opened the door to the influence of the ‘anti-vaxx’ movement and in the first half of 2019, the Philippines reported over 35 000 cases of measles, a virtually entirely vaccine preventable disease, and close to 500 deaths, a six-fold increase compared to 2018 (Board, 2019).

Besides applications, other forms of online media can also be breeding grounds for health misinformation. With social media platforms gaining traction, and even more so with the COVID-19 pandemic and its subsequent lockdowns, many health organizations and physicians are taking health education and treatment online. However, while there are qualified medical experts who provide reliable and accurate information on these platforms, health content is also generated by social influencers who are mostly not medically or professionally qualified. For instance, a study on YouTube videos revealed that the platform contained misleading information that were yet to be approved by the appropriate agencies (Madathil *et al.*, 2015). In addition, these videos were still receiving an increasing number of views despite medically unverified content.

How do we address this? There are lessons from the COVID-19 pandemic. The World Health Organization has offered a six-point framework aimed at governments and policy makers to help mitigate the COVID-19 infodemic which coupled with Eysenbach’s ‘4-Pillar’ approach comprising (i) information monitoring, (ii) building eHealth and science literacy capacity, (iii) encouraging fact checking and peer-review and (iv) accurate and timely knowledge translation, whilst minimizing distorting factors. could be adapted for combating similar issues in DHP. Beyond this, governments should also recognize that misinformation, especially among those with lower levels of literacy, can be a problem (Diviani *et al.*, 2015), and seek to implement a more tailored approach in educating different segments of the population regarding ways to objectively evaluate the quality of health information (Sun *et al.*, 2019).

### Ethics of influencing—limits of ‘nudging’ and the right of free choice

To what extent does nudging become coercing? As per Thaler and Sunstein earlier (2008, p. 6), a nudge is ‘any aspect of the choice architecture that alters people’s behavior in a predictable way without forbidding any options or significantly changing their economic incentives. To count as a mere nudge, the intervention must be easy and cheap to avoid. Nudges are not mandates’. (Thaler and Sunstein, 2008). However, nudging can become manipulation or coercion if used with the wrong intentions, becoming ethically unacceptable, which may result in unwanted backlash from the public. Professor Mike Kelly, United Kingdom Public Health leader, also acknowledges that ‘the counter argument [to the benefits of nudging] is that people are being nudged to buy sugary drinks and fattening food in an obesogenic environment that nudges them to eat at every opportunity and no one’s consent has been sought for that, so that’s just as unethical’ (Sax Institute, 2015). Note that the ethics around influencing are not unique to digital nudges, but digital nudges can be greatly scaled up at low cost as described above and hence the reach far outstrips that of conventional approaches.

To address the ethical concerns of nudging, a European Conference Information Systems research paper suggested three considerations (Lembcke *et al.*, 2019) nudgers should take into account. They are: (i) preserving freedom of choice and autonomy of individuals; (ii) ensuring transparency; (iii) aligning nudger’s goals with individuals in order to render a nudge as justifiable.

Ultimately, cultural differences will shape how far societies want to go when it comes to nudging including digital nudging in health promotion. It is thus crucial that public health agencies engage the wider society in such discussions about norms and ethical boundaries even as digital nudging becomes more and more used in health promotion.

### Minimizing social inequities through widespread accessibility and affordability

Without adequate attention to ensuring access and affordability, the benefits of DHP may only reach the privileged in society. Internet access remains unequal both across and within countries (Conceição, 2019) and these continue downstream into access to digital programs and interventions to improve one’s health.

Studies have shown that digital divide is still a major issue across different continents of the world especially among traditionally hard-to-reach populations such as those who are older, illiterate, lower in educational attainment, and rural areas with poorer infrastructure (Bol *et al.*, 2018; Labrique *et al.*, 2018; Estacio *et al.*, 2019). In addition, challenges remained in low-resourced settings to bring appropriate content and customize them into local languages that are digestible to the targeted populations (Royston *et al.*, 2015). Governments have to recognize these and proactively and preferentially fund not just physical access to appropriate devices but also the enabling connectivity. For example, references can be made to a review that laid out the 18 core principles in a framework known as 'Digital Universal Precautions’ as a mandate for healthcare organizations when promoting e-Health literacy among the citizens (Smith and Magnani, 2019).

This is a matter of urgency as disadvantaged groups are likely to become even more disadvantaged over time as more and more health promotion content and programming goes online and correspondingly minimized offline. A 2016 Survey of Adult Skills conducted by OECD found that a significant proportion of adults not only had poor reading skills (18.5%) and poor numeracy skills (22.7%), but one in four adults also had no or very limited experiences with digital devices such as computers.

Interventions have to be along three dimensions: (i) enabling all individuals to become digitally competent and confident enough to benefit, (ii) ensuring that digital content access and meaningful use are designed for those with minimal digital literacy and (iii) investing sufficiently in offline resources so that the population segments which cannot bridge this digital divide still have some means to access health promotion.

## Conclusion

Digitalization is sweeping across the entire world with the process accelerated by the ongoing COVID-19 pandemic. This ‘digitalization of everything’ has brought many benefits to the world including vastly expanding access to information and services but also raised the unhappy spectre of social media radicalization, easier identification and targeting of minorities and disadvantaged populations as well as worsening equity from deeper digital divides between the haves and have-nots. Health and healthcare services will continue to digitalize whatever we do. We have an opportunity to learn about these powerful forces and how to harness them for good. We have described many examples in this article of how DHP is enabling and can enable more people to access and benefit from knowledge and interventions to live their healthiest possible lives. We have also highlighted that the current reality of ‘caveat emptor’ with most available digital health applications released with minimal or no evaluation is most unsatisfactory. Finally, we have also highlighted the risks of unfettered and irresponsible use of digital technologies in health promotion including worsening inequities, cybersecurity lapses and inappropriate manipulation of unsuspecting persons. Whether DHP lives up to its promise or descends to the lowest depths of peril is in our hands, today’s public health leaders.

## DISCLAIMER

The authors alone are responsible for the views expressed in this article and they do not necessarily represent the views, decisions or policies of the institutions with which they are affiliated.
